# Supplementary material for: A genome-wide association study identifies the GPM6A locus associated with age at onset in ALS
Source: Commun Biol. 2025 Dec 5;8:1720. doi: 10.1038/s42003-025-09168-4 (PMC12680644; doi:10.1038/s42003-025-09168-4)
Supplement: Supplementary file 2 — Supplementary Information [file 42003_2025_9168_MOESM2_ESM.pdf]

Supplementary Materials

A genome-wide association study identifies the *GPM6A* locus associated with age at onset in ALS

Contents

1. Supplementary Figures 1–7

2. Supplementary Tables 1–8

3. Supplementary Note 1. Members of the Japanese Consortium for Amyotrophic Lateral Sclerosis research (JaCALS)

4. Supplementary References

a) b)

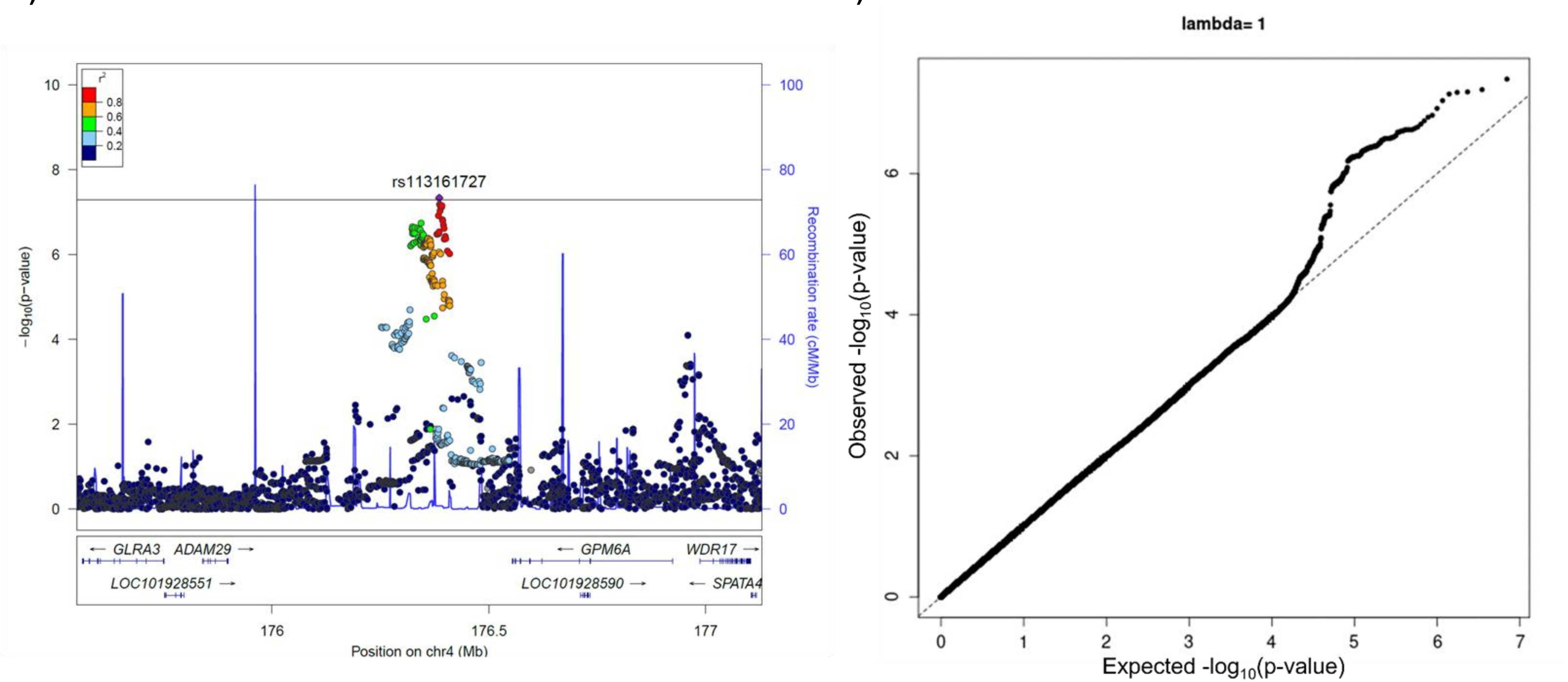

Supplementary Figure 1. Regional association plots for the 4q34.2 locus and quantile-quantile plot for the p-values in the discovery cohort

(a) Regional association plots for the 4q34.2 locus identified in the discovery cohort. The vertical axis represents $-\log_{10}$ (p-value) for the assessment of the association between each SNP and age at onset. Colors indicate the linkage disequilibrium (r^2) between each sentinel SNP and neighboring SNPs based on the JPT population of the 1000 Genomes Project phase 3. (b) Quantile-quantile plot (Q-Q plot) for the p-values in the discovery cohort. The vertical and horizontal axes indicate the observed and expected $-\log_{10}$ (p-value) for tests of association between single-nucleotide polymorphisms and AAO of Japanese patients with

ALS, respectively. Abbreviations: SNP, single-nucleotide polymorphism; JPT, Japanese people in Tokyo, Japan; ALS, amyotrophic lateral sclerosis; AAO, age at onset.

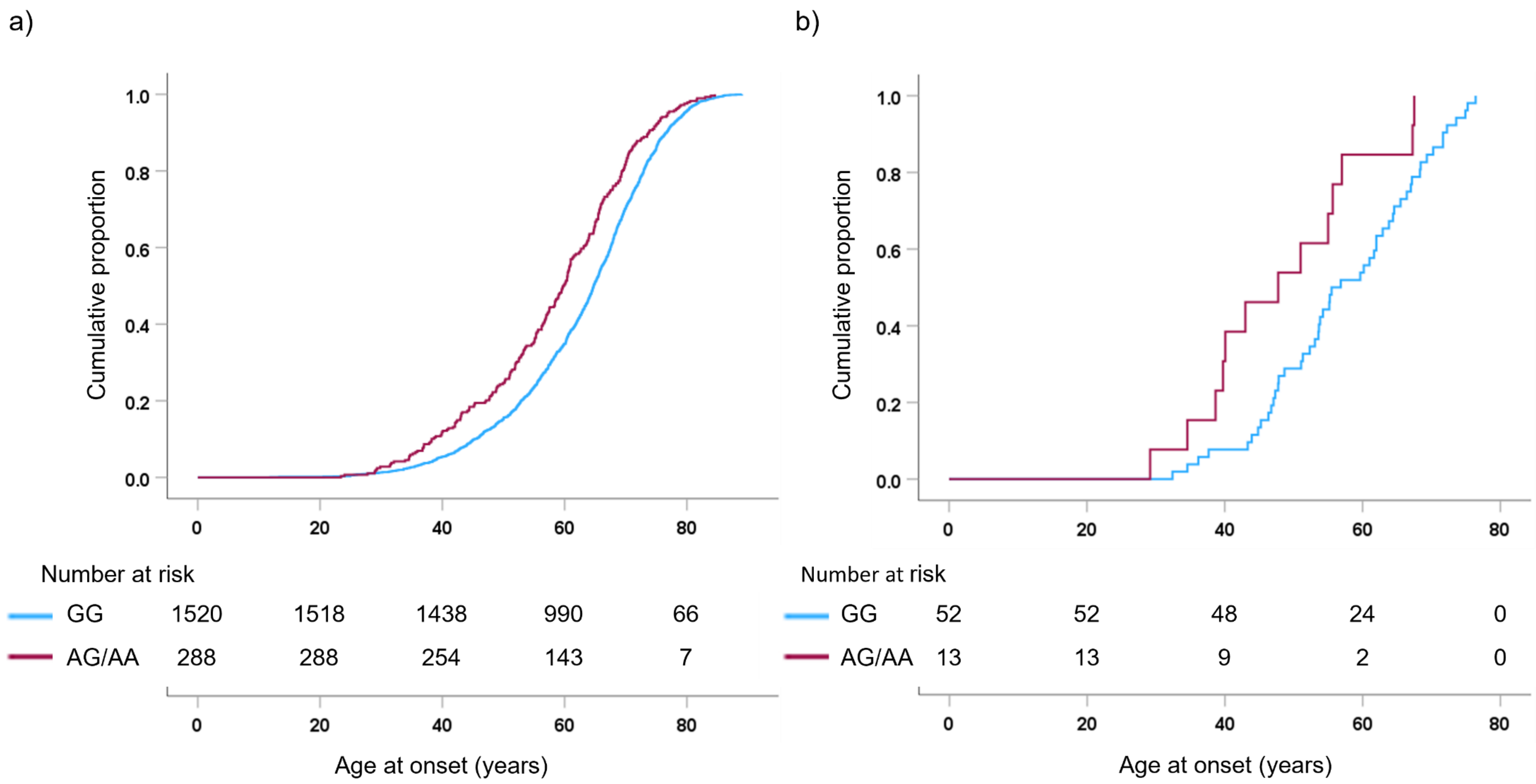

Supplementary Figure 2. Cumulative distribution of age at onset in Japanese patients with ALS stratified by rs113161727 genotype

Cumulative distribution of age at onset in Japanese patients with ALS (a) in the discovery cohort and (b) in patients with *SOD1*-ALS, stratified by rs113161727 genotype. Abbreviations: ALS, amyotrophic lateral sclerosis.

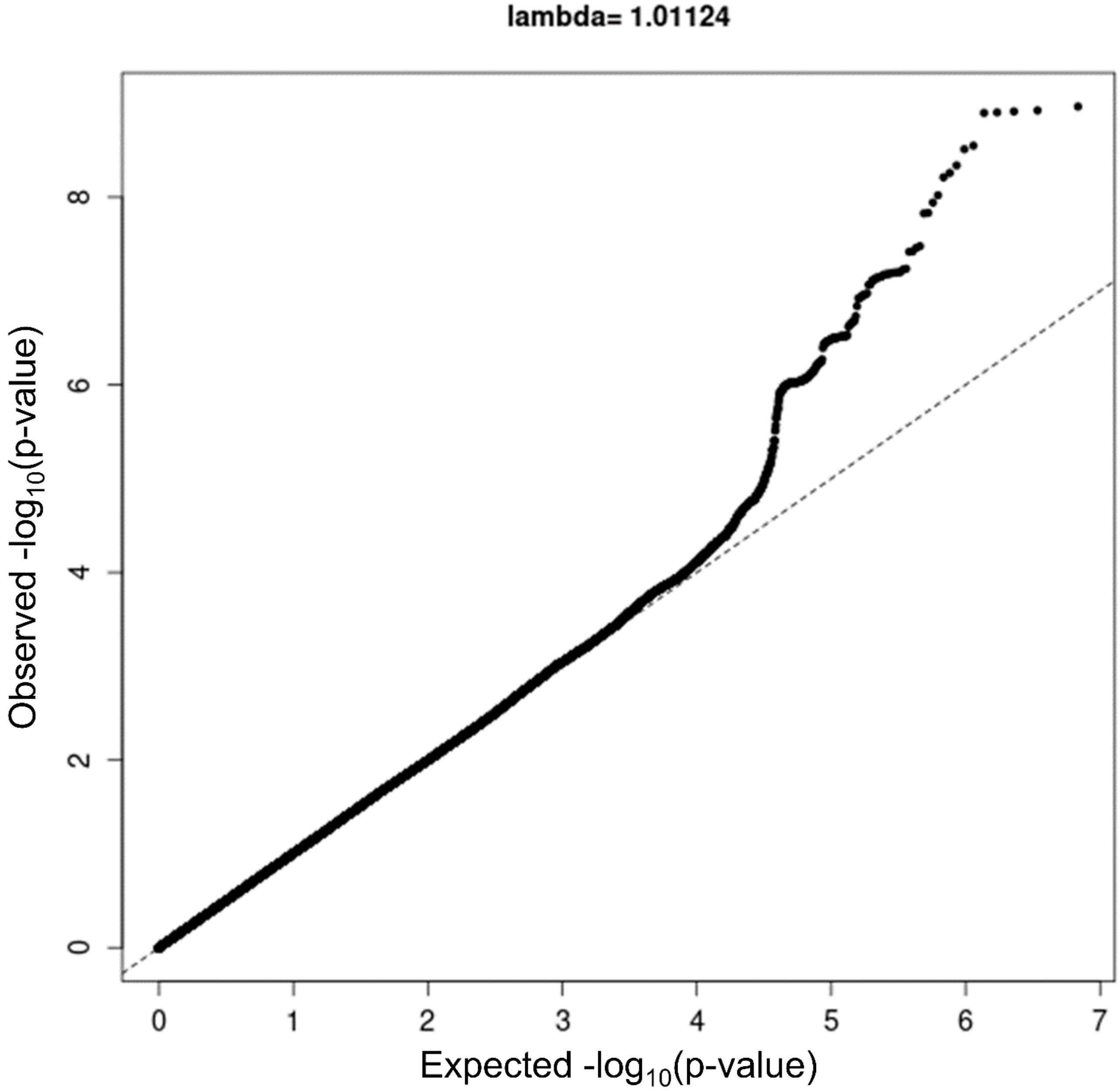

Supplementary Figure 3. Q-Q plot of the meta-analysis for the age at onset in Japanese patients with ALS

The vertical and horizontal axes indicate the observed and expected $-\log_{10}$ (p-value) for tests of association between single-nucleotide polymorphisms and age at onset of Japanese patients with ALS, respectively. Abbreviations: ALS, amyotrophic lateral sclerosis; Q-Q plot, quantile-quantile plot.

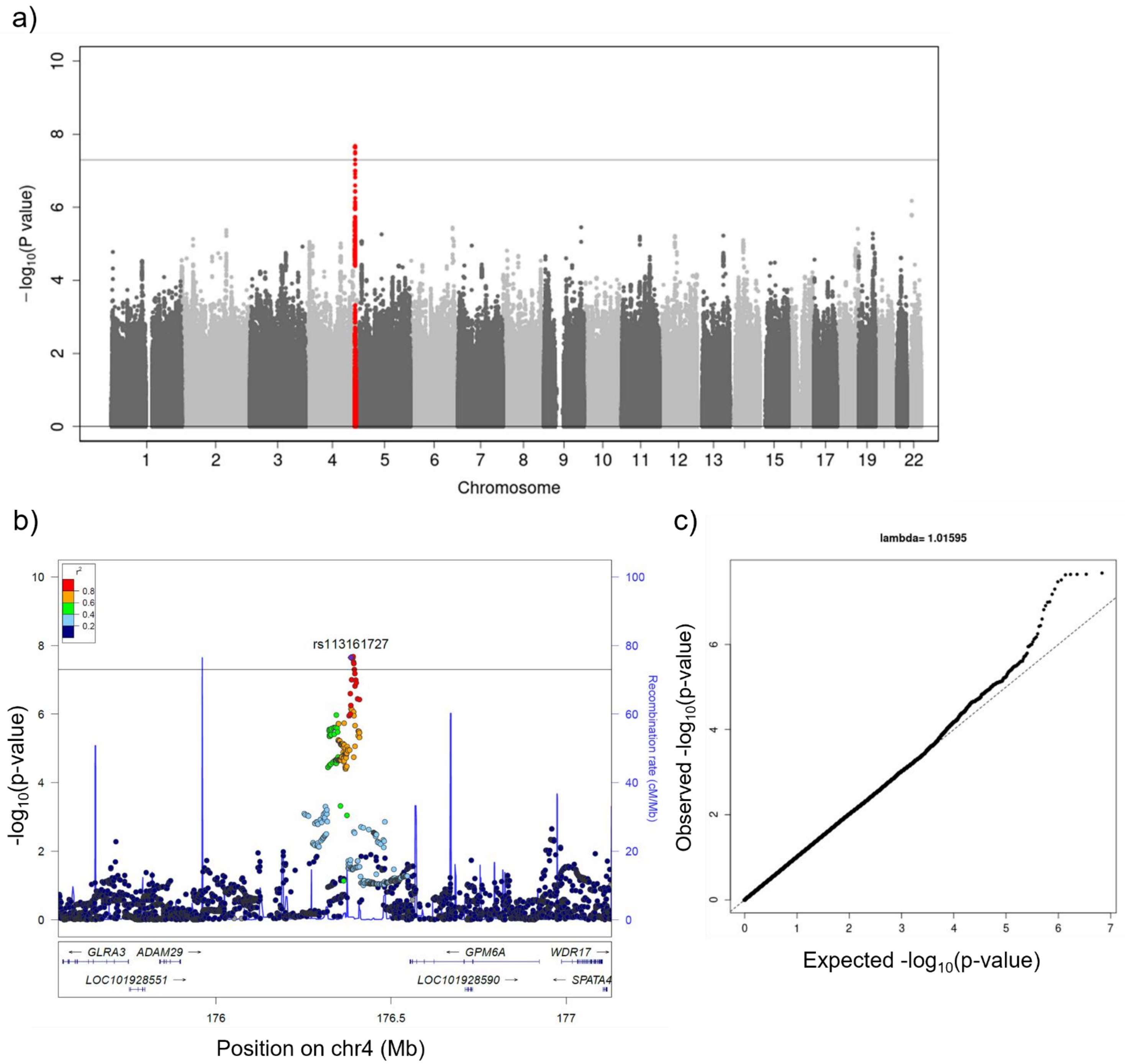

Supplementary Figure 4. Genome-wide meta-analysis for AAO in ALS patients without *SOD1* mutation

(a) Manhattan plot of the meta-analysis for AAO in Japanese ALS patients without *SOD1* mutation.

The results from the discovery and replication cohorts, excluding patients with *SOD1*-ALS, were combined in a meta-analysis. The meta-analysis confirmed the genome-wide significance of SNPs at the 4q34.2 locus.

(b) Regional association plots for the 4q34.2 locus identified in the meta-analysis. The vertical axis represents -$\log_{10}$ (p-value) for assessing the association between each SNP and AAO. Colors indicate the linkage disequilibrium (r^2) between each neighboring SNP and rs113161727 based on the JPT population of the 1000

Genomes Project Phase 3. The SNP rs113161727 represents the lead SNP in the meta-analysis which includes all patients, but not in the analysis excluding patients with *SOD1*-ALS (Supplementary Table 5).
(c) Q-Q plot for the p-values in the meta-analysis. The vertical and horizontal axes indicate the observed and expected $-\log_{10}$ (p-value) for tests of association between the SNPs and AAO in patients with ALS, respectively.
Abbreviations: SNP, single-nucleotide polymorphism; JPT, Japanese people in Tokyo, Japan; ALS, amyotrophic lateral sclerosis; AAO, age at onset

a)

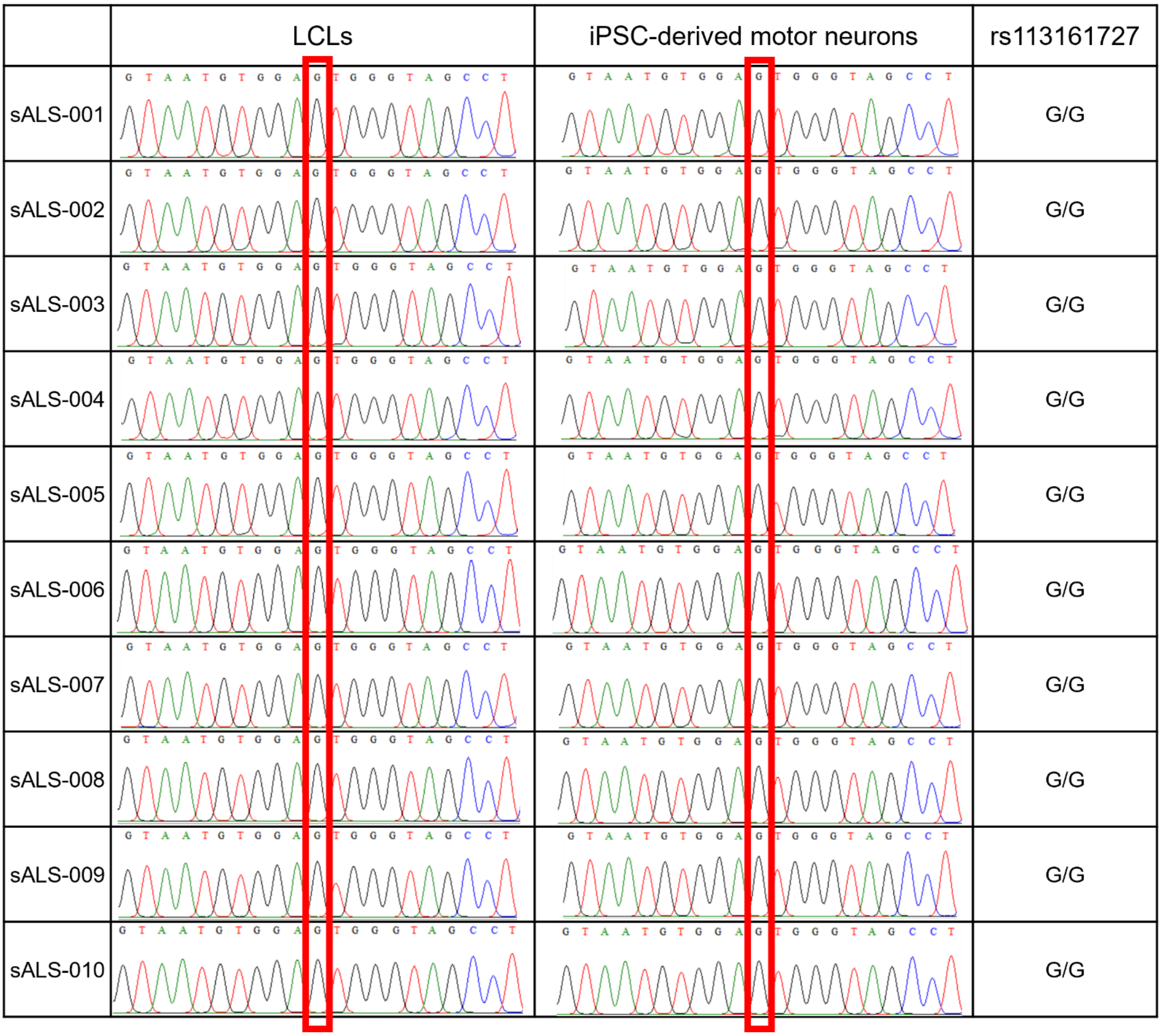
LCLs
iPSC-derived motor neurons
rs113161727
sALS-001
G T A A T G T G G A G T G G G T A G C C T
G T A A T G T G G A G T G G G T A G C C T
G/G
sALS-002
G T A A T G T G G A G T G G G T A G C C T
G T A A T G T G G A G T G G G T A G C C T
G/G
sALS-003
G T A A T G T G G A G T G G G T A G C C T
G T A A T G T G G A G T G G G T A G C C T
G/G
sALS-004
G T A A T G T G G A G T G G G T A G C C T
G T A A T G T G G A G T G G G T A G C C T
G/G
sALS-005
G T A A T G T G G A G T G G G T A G C C T
G T A A T G T G G A G T G G G T A G C C T
G/G
sALS-006
G T A A T G T G G A G T G G G T A G C C T
G T A A T G T G G A G T G G G T A G C C T
G/G
sALS-007
G T A A T G T G G A G T G G G T A G C C T
G T A A T G T G G A G T G G G T A G C C T
G/G
sALS-008
G T A A T G T G G A G T G G G T A G C C T
G T A A T G T G G A G T G G G T A G C C T
G/G
sALS-009
G T A A T G T G G A G T G G G T A G C C T
G T A A T G T G G A G T G G G T A G C C T
G/G
sALS-010
G T A A T G T G G A G T G G G T A G C C T
G T A A T G T G G A G T G G G T A G C C T
G/G

b)

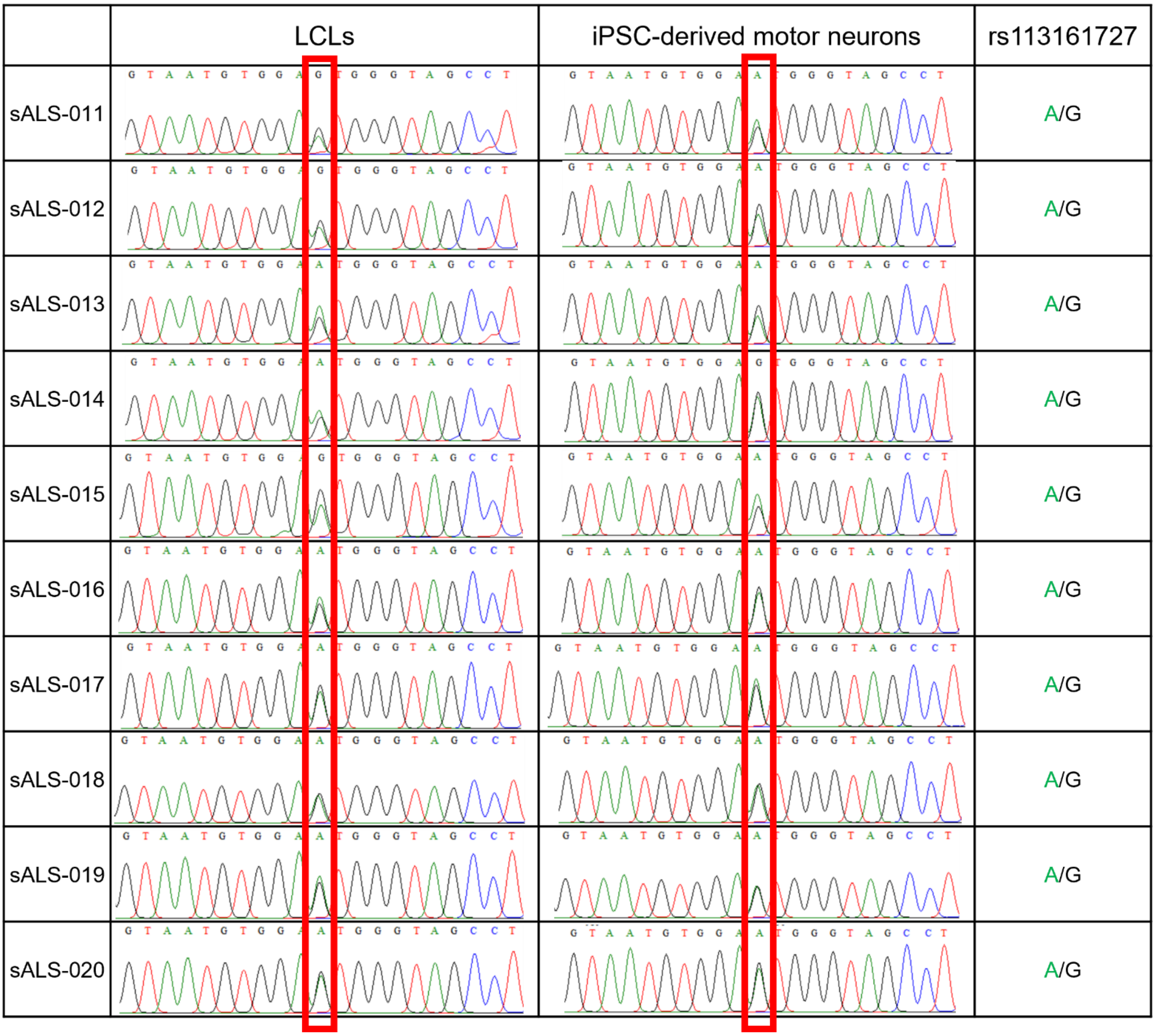

Supplementary Figure 5. Confirmation of rs113161727 genotypes in LCLs and iPSC-derived motor neurons by Sanger sequencing

Sequencing chromatograms showing the rs113161727 genotypes in LCLs and iPSC-derived motor neurons from patients with ALS used for RT-qPCR.

(a) Sequencing chromatograms from patients with the GG genotype confirm homozygosity at the rs113161727 locus in both cell types. (b) Sequencing chromatograms from patients with the AG genotype confirm the presence of both alleles at the rs113161727 locus in LCLs and iPSC-derived motor neurons.

Abbreviations: LCLs, lymphoblastoid B-cell lines; iPSC, induced pluripotent stem cell; RT-qPCR, real-time quantitative reverse transcription-polymerase chain reaction.

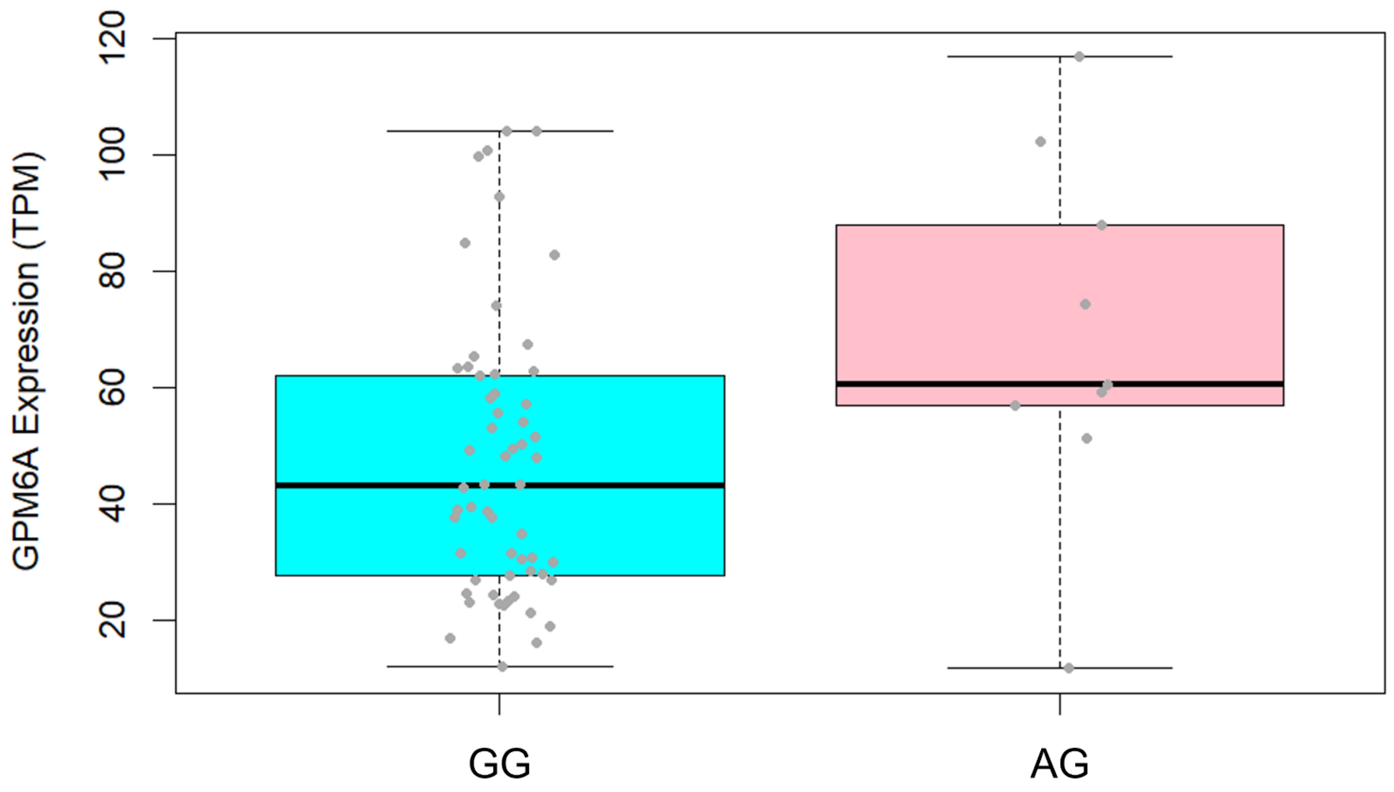

Supplementary Figure 6. *GPM6A* expression stratified by rs113161727 genotype in iPSC-derived motor neurons from patients with ALS.

Box plot displaying *GPM6A* transcript levels (TPM) obtained from RNA sequencing of induced pluripotent stem cell (iPSC)-derived motor neurons from patients with ALS, stratified by rs113161727 genotype. Each gray dot represents an individual sample (AG: n = 9; GG: n = 58). The bottom and top of the box indicate the interquartile ranges (25th and 75th percentiles), and the line represents the median. Whiskers under and over the box correspond to a 1.5 × interquartile range. *GPM6A* expression was significantly higher in the AG genotype than in the GG genotype (p = 0.029, Mann–Whitney *U* test).

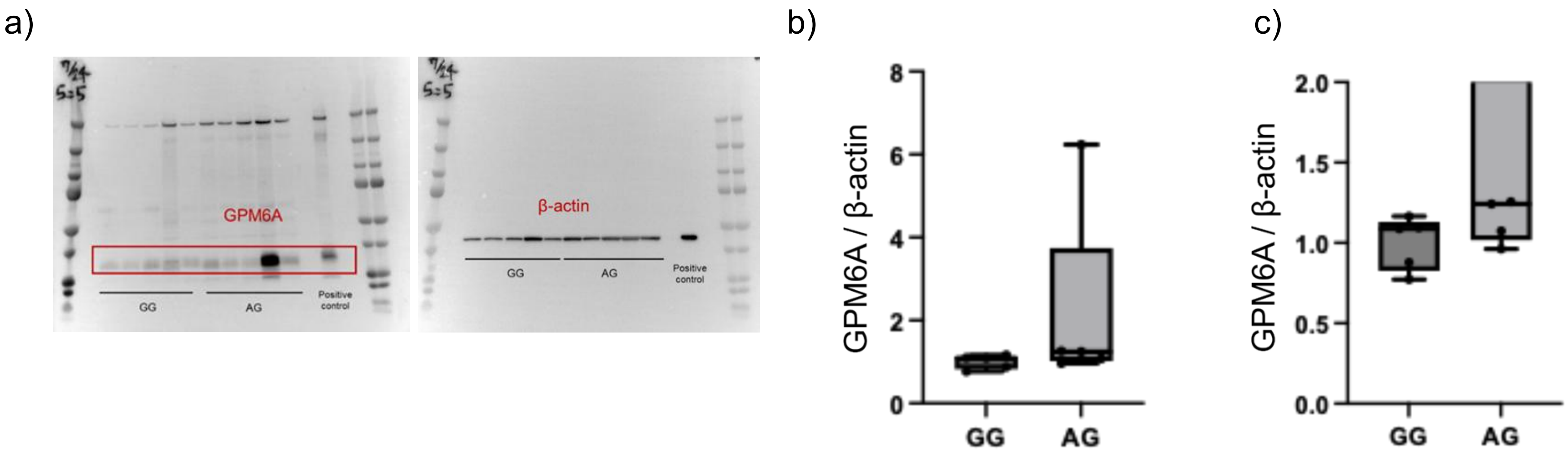

Supplementary Figure 7. Western blotting of GPM6A in iPSC-derived motor neurons stratified based on rs113161727 genotype.

(a) Uncropped immunoblots of GPM6A and the loading control β-actin using iPSC-derived motor neurons from five AG and five GG carriers. SH-SY5Y cells transfected with GPM6A-GFP were used as a positive control. Molecular weight markers are shown for all blots. (b) Box plot showing GPM6A protein expression in iPSC-derived motor neurons from five patients in each genotype group. (c) Magnified view of Supplementary Figure 7b. Band intensities were quantified using ATTO Densitograph software (ATTO, Tokyo, Japan), normalized to β-actin, and expressed relative to the mean value of the GG group, which was set to 1. The primary antibody for GPM6A was anti-GPM6A (Thermo Fisher Scientific #720252), and the loading control antibody was anti-β-actin (Cell Signaling Technology #4970). Comparison between genotype groups was conducted using the Mann–Whitney *U* test. Although higher GPM6A protein expression was observed in some AG genotype cases, the difference was not statistically significant ($p = 0.22$). The bottom and top of the box indicate the interquartile range (25th and 75th percentiles) and the line represents the median. The whiskers under and over the box represent to the minimum and maximum values, respectively.

Supplementary Table 1. Clinical features of the patients included in the study

	**Discovery cohort**	**Replication cohort**	**Total**	**P-value**
Number of patients	1808	207	2015	
Age at onset (mean ± S.D.)	62.0±12.4	64.4±11.3	62.2±12.3	0.009
Sex (Male/Female)	1050/758	115/92	1165/850	0.487
Age at onset in males (mean ± S.D.)	61.2±12.4	63.9±11.3	61.5±12.3	0.026
Age at onset in females (mean ± S.D.)	63.0±12.4	64.9±11.2	63.2±12.3	0.164
Median survival time from onset (years, IQR)	3.8 (2.2-7.7)	4.5 (2.3-7.1)	3.8 (2.2-7.7)	0.818

S.D., Standard deviation; 95%CI, 95% confidence interval.

P-values represent statistical comparisons between the discovery cohort and the replication cohort. Comparisons of age at onset were performed using Student's t-test, and comparisons of sex ratios were conducted using the $\chi 2$ test. The median survival time was estimated using the Kaplan-Meier method and compared using the log-rank test.

Supplementary Table 2. Clinical features of ALS patients with or without a *SOD1* mutation included in the study

	Discovery cohort			Replication cohort	
	**Patients without a *SOD1* mutation**	**Patients with *SOD1*-ALS**	**p-value**	**Patients without a *SOD1* mutation**	**p-value**
Number of patients	1743	65		206	
Age at onset (years, mean ± S.D.)	62.2±12.4	55.4±12.0	1.36×10^{-5}	64.4±11.3	8.30×10^{-8}
Sex ratio (Male/Female)	1.39 (1015/728)	1.17 (35/30)	0.482	1.24 (114/92)	0.833
Age at onset in males (mean ± S.D.)	61.3±12.4	58.6±10.4	0.203	64.0±11.3	0.014
Age at onset in females (mean ± S.D.)	63.5±12.2	51.7±12.8	2.65×10^{-5}	64.9±11.2	3.40×10^{-7}
Median survival time from onset (years, 95%CI)	3.8 (3.5-4.0)	7.7 (4.0-11.4)	8.14×10^{-4}	4.5 (3.7-5.3)	0.017

S.D., standard deviation; 95%CI, 95% confidence interval.

The p-values in the left column represent statistical comparisons within the discovery cohort (patients with vs without a *SOD1* mutation). The p-values in the right column represent statistical comparisons between patients with *SOD1*-ALS in the discovery cohort and patients without a *SOD1* mutation in the replication cohort. Statistical comparisons involving the *SOD1*-ALS patient in the replication cohort were not performed, as there was only one patient, making statistical analysis infeasible. Comparisons of age at onset were performed using Student's t-test, and comparisons of sex ratios were conducted using the $\chi 2$ test. The median survival time was estimated using the Kaplan–Meier method and compared using the log-rank test.

Supplementary Table 3. Genetic variants, age at onset, and rs113161727 genotype distribution in 65 patients with *SOD1*-ALS

Nucleotide change	Amino acid change	Number of patients	Age at onset (years, mean ± S.D.)	Number of patients by rs113161727 genotype	
				AA/AG	GG
c.10A>G	p.Lys4Glu	2	50.0±3.2	1	1
c.14C>A	p.Ala5Asp	1	76.4		1
c.20G>A	p.Cys7Tyr	3	63.0±8.1		3
c.25C>G	p.Leu9Val	1	62.9		1
c.37_54dup18	p.Gly13_Ile18dup	1	51.1	1	
c.44T>C	p.Val15Ala	2	69.6±2.9	1	1
c.116T>G	p.Leu39Arg	1	55.7	1	
c.131A>G	p.His44Arg	3	60.9±15.5	1	2
c.140A>G	p.His47Arg	4	42.9±9.8	1	3
c.280G>A	p.Gly94Ser	14	52.9±9.4	3	11
c.281G>T	p.Gly94Val	1	47.8		1
c.319C>G	p.Leu107Val	4	41.9±9.1		4
c.335G>A	p.Cys112Tyr	1	54.3		1
c.341T>C	p.Ile114Thr	2	63.7±3.8		2
c.380T>C	p.Leu127Ser	14	59.5±12.8	3	11
c.404G>A	p.Ser135Asn	6	56.1±12.9	1	5
c.425G>A	p.Gly142Glu	1	67.2		1
c.425G>C	p.Gly142Ala	1	61.7		1
c.435delGinsCGTTTA	p.Leu145Phefs*3	1	40.1		1
c.437C>T	p.Ala146Val	1	60.2		1
c.449T>C	p.Ile150Thr	1	37.7		1
Total		65	55.4±12.0	13	52

Position is based on Human Genome Assembly build 37.

S.D., standard deviation

Supplementary Table 4. Effect of rs113161727 among ALS patients with or without a *SOD1* mutation in the discovery cohort

SNP	Chr	Position	Gene	Alleles		Group	ALT frq.	Effect	SE	P-value
				REF	ALT					
rs113161727	4	176385733	*ADAM29, GPM6A*	G	A	*SOD1* non-carrier	0.083	-3.927	0.798	8.70E-07
						SOD1-ALS patients	0.108	-10.237	3.082	0.002

Position is based on Human Genome Assembly build 37.
SNP, single-nucleotide polymorphism; Chr, chromosome; REF, reference allele; ALT, alternative allele; frq, frequency; SE, standard error; HetPVal, p-value from the test of heterogeneity.

Supplementary Table 5. Genome-wide significant SNPs at 4q34.2 in the meta-analysis for the age at onset of ALS patients without *SOD1* mutation

SNP	Chr	Position	Gene	Rsq	Alleles		Meta-analysis					
					REF	ALT	ALT frq.	Effect	SE	p-value	I^2	HetPVal
rs9790521	4	176392011	*ADAM29, GPM6A*	0.967	G	A	0.918	4.002	0.714	2.10E-08	0	0.451
rs113161727	4	176385733	*ADAM29, GPM6A*	0.908	G	A	0.083	-4.106	0.734	2.23E-08	0	0.56
rs10461336	4	176391372	*ADAM29, GPM6A*	0.999	C	T	0.081	-3.931	0.703	2.25E-08	0	0.43
rs62336886	4	176386532	*ADAM29, GPM6A*	0.956	G	A	0.083	-3.997	0.715	2.26E-08	0	0.473
rs10033608	4	176388155	*ADAM29, GPM6A*	0.975	G	T	0.082	-3.963	0.709	2.31E-08	0	0.452
rs7699269	4	176393194	*ADAM29, GPM6A*	0.981	G	A	0.079	-3.976	0.718	3.05E-08	0	0.461
rs13136344	4	176393932	*ADAM29, GPM6A*	0.979	T	A	0.921	3.976	0.72	3.37E-08	0	0.465

Position is based on Human Genome Assembly build 37.
SNP, single-nucleotide polymorphism; Chr, chromosome; REF, reference allele; ALT, alternative allele; frq, frequency; SE, standard error; HetPVal, p-value from the test of heterogeneity.

Supplementary Table 6. Associations of previously reported SNPs associated with AAO in European or Chinese patients with ALS

SNP	Locus	Chr	Position	Gene	Function	Imputed /Genotyped	Rsq	Alleles		ALT frq. of 1000 genomes phase 3					Meta-analysis						Discovery cohort				Replication cohort			
								REF	ALT	AFR	AMR	EAS	EUR	SAS	ALT frq.	Effect	SE	p-value	I^2	HetPVal	ALT frq.	Effect	SE	p-value	ALT frq.	Effect	SE	p-value
rs3011225 [1]	1p34.1	1	44319373	*ST3GAL3*	intronic	Genotyped	1.000	G	A	0.180	0.510	0.720	0.740	0.550	0.629	0.076	0.400	0.850	0	0.602	0.632	-0.003	0.428	0.990	0.611	0.628	1.132	0.580
rs803675 [1]	1p34.1	1	44353237	*ST3GAL3*	intronic	Imputed	0.991	G	A	0.520	0.650	0.730	0.750	0.550	0.655	0.302	0.410	0.461	0	0.512	0.658	0.200	0.438	0.650	0.633	1.012	1.158	0.383
rs10128627 [2]	11q13.1	11	65170600	*FRMD8*	intronic	Imputed	0.978	T	C	0.091	0.340	0.068	0.160	0.098	0.088	0.597	0.686	0.384	0	0.943	0.085	0.576	0.743	0.440	0.104	0.715	1.785	0.689
rs2046243 [3]	18q21.1	18	46290524	*CTIF*	intronic	Imputed	0.928	A	G	0.990	0.760	0.890	0.770	0.720	0.840	-0.393	0.538	0.466	0	0.946	0.839	-0.379	0.576	0.510	0.849	-0.489	1.508	0.746

rs6801884 [2] was excluded from the analysis owing to its imputation quality score < 0.8.

Position is based on Human Genome Assembly build 37.
SNP, single-nucleotide polymorphism; Chr, chromosome; REF, reference allele; ALT, alternative allele; frq, frequency; AFR, African; AMR, Admixed American; EAS, East Asian; EUR, European; SAS, South Asian; SE, standard error; HetPVal, p-value from the test of heterogeneity.

Supplementary Table 7. Primer sequences for real-time quantitative reverse transcription-polymerase chain reaction

Gene symbol	Name	Forward primer	Reverse primer
GPM6A	glycoprotein M6A	5′- ACCTTGACGCTCTTCTTTTCATTATT -3′	5′- CAATGGTCAAATCAGTGCACAA -3′
ADAM29	ADAM metallopeptidase domain 29	5′- GTCATGACCGCAATGAACAG -3′	5′- TGACCAACACGGTCACCTAA -3′
GLRA3	glycine receptor alpha 3	5′- GGCTCTATCGCAGAGACGAC -3′	5′- GGAGGGGTCGAGGTCTAAAG -3′
WDR17	WD repeat domain 17	5′- GCATAGCCATTGGTGATGTG -3′	5′- AGGCACGGAAACATGTAAGG -3′
SPATA4	spermatogenesis-associated protein 4	5′- CGTCACTTTCGCCACAGCTA -3′	5′- AGGAAGCCATTTGAAAATCTCTGT -3′
ACTB	actin beta	5′- TGAAGTGTGACGTGGACATC-3′	5′- TGAAGTGTGACGTGGACATC-3′

Supplementary Table 8. Antibodies used for western blotting

Antibody	Host and clonality	Manufacturer	Catalog number	RRID
GPM6A	Rabbit polyclonal	Thermo Fisher Scientific	#720252	AB_2633216
β-actin	Rabbit monoclonal, clone 13E5	Cell Signaling Technology	#4970	AB_2223172
Secondary HRP-Conjugated anti-rabbit IgG	Donkey polyclonal, HRP-conjugated $F(ab')_2$	Cytiva	#NA9340	AB_772191

Supplementary Note 1.

Members of the Japanese Consortium for Amyotrophic Lateral Sclerosis research (JaCALS)

JaCALS members included Drs Gen Sobue, Naoki Atsuta, Manabu Doyu, Jun-ichi Niwa, Ryoichi Nakamura, Genki Tohnai (Aichi Medical University), Daisuke Ito, Naoki Hayashi, Daichi Yokoi, Hazuki Watanabe, Jo Senda, Mizuki Ito, Hirohisa Watanabe, Masahisa Katsuno (Nagoya University, Site Investigator), Mitsuya Morita (Jichi Medical University, Site Investigator), Naoki Suzuki, Masashi Aoki (Tohoku University, Site Investigator), Tomohiko Ishihara, Osamu Onodera (Niigata University, Site Investigator), Kota Bokuda, Toshio Shimizu, Akihiro Kawata (Tokyo Metropolitan Neurological Hospital, Site Investigator), Yoshiaki Yamamoto, Tomokazu Obi (Shizuoka Institute of Epilepsy and Neurological Disorders, Site Investigator), Rina Hashimoto, Ikuko Aiba (National Hospital Organization Higashinagoya National Hospital, Site Investigator), Miho Akaza, Takanori Yokota (Institute of Science Tokyo, Site Investigator), Hiroya Naruse, Tatsushi Toda (Tokyo University, Site Investigator), Kazuko Hasegawa (National Hospital Organization Sagamihara National Hospital, Site Investigator), Asako Tamura, Akira Taniguchi (Mie University, Site Investigator), Yuishin Izumi, Ryuji Kaji (Tokushima University, Site Investigator), Seiya Noda, Satoshi Kuru (National Hospital Organization Suzuka National Hospital, Site Investigator), Takuji Fujita (Takumi-kai Neurology Clinic, Site Investigator), Masahiro Wakita, Ikuko Iwata, Ichiro Yabe (Hokkaido University, Site Investigator), Masayuki Sato, Yukio Fujita, Yoshio Ikeda (Gumma University, Site Investigator), Arifumi Matsumoto, Hiroaki Ito, Isao Nagano (National Hospital Organization Miyagi National Hospital, Site Investigator), Masaya Oda (Vihara Hananosato Hospital, Site Investigator), Atsuko Tsuneyama, Yuji Takahashi (National Center Hospital, National Center of Neurology and Psychiatry, Site Investigator), Toru Yamashita, Hiroyuki Ishiura (Okayama University, Site Investigator), Takamasa Kitaoji, Yuichi Noto, Toshiki Mizuno (Kyoto Prefectural University, Site Investigator), Tomoko Nakazato, Nobutaka Hattori (Juntendo University, Site Investigator), Takashi Ayaki, Ryosuke Takahashi (Kyoto University, Site

Investigator), Takehisa Hirayama, Osamu Kano (Toho University, Site Investigator), Kazumasa Shindo, Takamura Nagasaka(Yamanashi University, Site Investigator), Yasuhiro Watanabe, Ritsuko Hamajima (Tottori University, Site Investigator), Mie Nakamura (National Hospital Organization Tokyo National Hospital, Site Investigator), Senri Ko, Yu Hashimoto, Ryo Yamasaki (Kyushu University, Site Investigator), Kazumoto Shibuya, Satoshi Kuwabara (Chiba University, Site Investigator), Hiroo Terashi, Haruhisa Kato, Hitoshi Aizawa (Tokyo Medical University, Site Investigator), Yuichi Higashiyama, Hiroshi Doi, Fumiaki Tanaka (Yokohama City University, Site Investigator), Kouichi Mizoguchi (National Hospital Organization Shizuoka Medical Center, Site Investigator), Kohei Suzuyama, Haruki Koike (Saga University, Site Investigator), Kazuaki Kanai (Fukushima Medical University, Site Investigator), Chiho Ishida, Kiyonobu Komai (National Hospital Organization Iou National Hospital, Site Investigator), Nobuhiro Ogawa, Makoto Urushitani (Shiga University of Medical Science, Site Investigator), Yoshio Tsuboi (Fukuoka University, Site Investigator), Kensuke Ikenaka, Hideki Mochizuki (Osaka University, Site Investigator), Keiko Ohnari, Hiroaki Adachi (University of Occupational and Environmental Health, Site Investigator), Ryosuke Oki (Tonan Hospital, Site Investigator), Hideyuki Takeuchi (International University of Health and Welfare Atami Hospital, Site Investigator), Tatsuhiko Yuasa (Kamagaya General Hospital, member of the JaCALS steering committee), and Shoji Tsuji (Institute of Medical Genomics, International University of Health and Welfare, member of the JaCALS steering committee).

Supplementary References

1 Ahmeti, K. B. *et al.* Age of onset of amyotrophic lateral sclerosis is modulated by a locus on 1p34.1. *Neurobiol. Aging* **34**, 357.e7–19 (2013).

2 Li, C. *et al.* Genome-wide analyses identify NEAT1 as genetic modifier of age at onset of amyotrophic lateral sclerosis. *Mol. Neurodegener*. **18**, 77 (2023).

3 Li, C. *et al.* Genetic modifiers of age at onset for amyotrophic lateral sclerosis: a genome-wide association study. *Ann. Neurol*. **94**, 933–941 (2023).
