# Supplementary material for: A genome-wide association study identifies the GPM6A locus associated with age at onset in ALS
Source: Commun Biol. 2025 Dec 5;8:1720. doi: 10.1038/s42003-025-09168-4 (PMC12680644; doi:10.1038/s42003-025-09168-4)
Supplement: Supplementary file 3 — Description of Additional Supplementary Materials [file 42003_2025_9168_MOESM3_ESM.pdf]

Description of Additional Supplementary Files

File name: Supplementary Data 1

Description: All 18 genome-wide significant SNPs identified in the meta-analysis combining the discovery and replication cohorts

File name: Supplementary Data 2

Description: Source data for Figure 2 (distribution of age at onset and the relationship between AAO and rs113161727 genotype)

File name: Supplementary Data 3

Description: Source data for Figure 3 (association between rs113161727 and site of onset)

File name: Supplementary Data 4

Description: Source data for Figure 4 (relative expression levels of genes surrounding rs113161727 in iPSC-derived motor neurons from patients with ALS stratified by rs113161727 genotype)

File name: Supplementary Data 5

Description: Source data for Supplementary Figures 6
