# Supplementary material for: A genome-wide association study identifies the GPM6A locus associated with age at onset in ALS
Source: Commun Biol. 2025 Dec 5;8:1720. doi: 10.1038/s42003-025-09168-4 (PMC12680644; doi:10.1038/s42003-025-09168-4)
Supplement: Supplementary file 9 — Reporting Summary [file 42003_2025_9168_MOESM9_ESM.pdf]

nature portfolio

Corresponding author(s): Masahiro Nakatochi, Gen Sobue

Last updated by author(s): Oct 28, 2025

Reporting Summary

Nature Portfolio wishes to improve the reproducibility of the work that we publish. This form provides structure for consistency and transparency in reporting. For further information on Nature Portfolio policies, see our Editorial Policies and the Editorial Policy Checklist.

Statistics

For all statistical analyses, confirm that the following items are present in the figure legend, table legend, main text, or Methods section.

n/a	Confirmed	
☐	☒	The exact sample size (n) for each experimental group/condition, given as a discrete number and unit of measurement
☐	☒	A statement on whether measurements were taken from distinct samples or whether the same sample was measured repeatedly
☐	☒	The statistical test(s) used AND whether they are one- or two-sided<br>*Only common tests should be described solely by name; describe more complex techniques in the Methods section.*
☐	☒	A description of all covariates tested
☐	☒	A description of any assumptions or corrections, such as tests of normality and adjustment for multiple comparisons
☐	☒	A full description of the statistical parameters including central tendency (e.g. means) or other basic estimates (e.g. regression coefficient) AND variation (e.g. standard deviation) or associated estimates of uncertainty (e.g. confidence intervals)
☐	☒	For null hypothesis testing, the test statistic (e.g. F, t, r) with confidence intervals, effect sizes, degrees of freedom and P value noted<br>*Give P values as exact values whenever suitable.*
☒	☐	For Bayesian analysis, information on the choice of priors and Markov chain Monte Carlo settings
☒	☐	For hierarchical and complex designs, identification of the appropriate level for tests and full reporting of outcomes
☐	☒	Estimates of effect sizes (e.g. Cohen's d, Pearson's r), indicating how they were calculated

Our web collection on statistics for biologists contains articles on many of the points above.

Software and code

Policy information about availability of computer code

Data collection	No software was used.
Data analysis	PLINK version 1.90b5.1 was used for GWAS. SHAPEIT2, Minimac3 were used for genotype imputation. The association between AAO and SNPs was assessed using the BOLT-LMM version 2.4.1 in the discovery cohort and EPACTS in the replication cohort. LocusZoom version 1.4 was used for the regional plot. METAL version 2011-03-25 was used for meta-analysis of GWAS. Statistical analyses for mRNA expression analyses were conducted using Prism 10, Trimmomatic v0.39 , STAR v2.7.11b, and RSEM v1.3.1 were used for the bioinformatic analysis of RNA-seq data. All detailed parameters are described in the main text.

For manuscripts utilizing custom algorithms or software that are central to the research but not yet described in published literature, software must be made available to editors and reviewers. We strongly encourage code deposition in a community repository (e.g. GitHub). See the Nature Portfolio guidelines for submitting code & software for further information.

Data

Policy information about availability of data

All manuscripts must include a data availability statement. This statement should provide the following information, where applicable:

- Accession codes, unique identifiers, or web links for publicly available datasets
- A description of any restrictions on data availability
- For clinical datasets or third party data, please ensure that the statement adheres to our policy

The individual ALS genotyping dataset in Japanese is managed by the Japanese Consortium for ALS research (JaCALS) with informed consent. The summary statistics and the RNA-seq gene-level raw count matrix are available from one of the Japanese public repositories (Human Genetic Variation Database; HGVD). We already contacted to the office of Human Genetic Variation Database (HGVD) and confirmed that our summary statistics file will be publicly available (Accession ID: HGV0000025).

Human research participants

Policy information about studies involving human research participants and Sex and Gender in Research.

Reporting on sex and gender	Sex was determined based on genetic data and self-reported information. Samples with discrepancies between genetic and reported sex were excluded during the quality control (QC) process to ensure data integrity. Sex ratio and age at onset differences were reported in the baseline characteristics. This approach was implemented solely to maintain the accuracy and reliability of the dataset.
Population characteristics	All individuals included in the genome-wide association study (GWAS) analysis, after quality control (QC), were of Japanese ancestry.
Recruitment	The participants in the discovery cohort were recruited from the JaCALS study, a multicenter patient registry for ALS involving 42 institutions across Japan. The replication cohort consisted of patients with ALS recruited from two Japanese institutions, Jichi Medical University and Hokkaido University.
Ethics oversight	The Ethics Review Committee of Aichi Medical University School of Medicine approved this study (approval number: 2021-083).

Note that full information on the approval of the study protocol must also be provided in the manuscript.

Field-specific reporting

Please select the one below that is the best fit for your research. If you are not sure, read the appropriate sections before making your selection.

☒ Life sciences ☐ Behavioural & social sciences ☐ Ecological, evolutionary & environmental sciences

For a reference copy of the document with all sections, see nature.com/documents/nr-reporting-summary-flat.pdf

Life sciences study design

All studies must disclose on these points even when the disclosure is negative.

Sample size	We used existing cohort data. Thus, no sample-size calculation was performed.<br>ALS is a rare neurodegenerative disease. Thus, we have used most of the ALS patients that were registered in JaCALS study (the largest ALS cohort in Japan; in this study 1,808 participants in total after QC.
Data exclusions	In the preprocessing step before GWAS, we excluded samples with a genotype call rate < 0.98. Samples with discrepancies between genetic and reported sex were also removed. Identity-by-descent (IBD) analysis detected duplicate or closely related sample pairs (PI_HAT > 0.1875), with one sample from each pair being excluded. Principal component analysis (PCA) with the 1000 Genomes Project reference panel (phase 3) identified individuals with estimated ancestries outside the Japanese population, who were subsequently removed.
Replication	To replicate our findings, we used an independent Japanese ALS samples from Hokkaido University and Jichi Medical University.
Randomization	Our study is an observational study. Thus, no randomization was performed.
Blinding	Our study is an observational study. Thus, no blinding was performed.

Reporting for specific materials, systems and methods

We require information from authors about some types of materials, experimental systems and methods used in many studies. Here, indicate whether each material, system or method listed is relevant to your study. If you are not sure if a list item applies to your research, read the appropriate section before selecting a response.

Materials & experimental systems

n/a	Involved in the study
☐	☒ Antibodies
☐	☒ Eukaryotic cell lines
☒	☐ Palaeontology and archaeology
☒	☐ Animals and other organisms
☐	☒ Clinical data
☒	☐ Dual use research of concern

Methods

n/a	Involved in the study
☒	☐ ChIP-seq
☒	☐ Flow cytometry
☒	☐ MRI-based neuroimaging

Antibodies

Antibodies used

GPM6A — rabbit polyclonal; Thermo Fisher Scientific, Cat# 720252, RRID: AB_2633216, dilution: 1:500
β-Actin (clone 13E5) — rabbit monoclonal; Cell Signaling Technology, Cat# 4970, RRID: AB_2223172, dilution: 1:1250
HRP-Conjugated anti rabbit IgG— rDonkey polyclonal, HRP conjugated $F(ab')_2$; Cytiva, Cat# NA9340, RRID: AB_772191, dilution: 1:5000

Validation

All primary antibodies used are commercially available and have been validated by the manufacturer. Supporting publications are found on the manufacturer's site; Specificity for GPM6A supported by a GPM6A-GFP positive control (SH-SY5Y). Uncropped blots with size markers are provided in Supplementary figure 7.

Eukaryotic cell lines

Policy information about cell lines and Sex and Gender in Research

Cell line source(s)

Human lymphoblastoid B cell lines (LCLs)
sALS-001,sALS-002, sALS-003, ..., and sALS-020 (ALS patients-derived LCLs)
All 20 sporadic ALS patients were registered in JaCALS.

Authentication

Full authentication (e.g., STR profiling) was not performed. However, confirmation of the rs113161727 genotype showed consistency with the corresponding patient's genotype.

Mycoplasma contamination

All cell lines were negative for mycoplasma contamination.

Commonly misidentified lines
(See ICLAC register)

No commonly misidentified cell lines were used in the study.

Clinical data

Policy information about clinical studies

All manuscripts should comply with the ICMJE guidelines for publication of clinical research and a completed CONSORT checklist must be included with all submissions.

Clinical trial registration

Our study is an observational study.

Study protocol

Our study is an observational study.

Data collection

In the discovery cohort, all patients with ALS are recruited from all regions in Japan. The detail of this project is written in Yokoi D et al (Journal of Neurology; 2016) The clinical data and DNA samples which we used in this study were collected from February, 2006 to February, 2022.

Outcomes

Identification of genetic variants associated with age at onset in Japanese patients with ALS
